# Genome-wide analysis of the cellulose synthase gene family in autopolyploid sugarcane (*Saccharum spontaneum*)

**DOI:** 10.3389/fpls.2025.1708654

**Published:** 2025-12-09

**Authors:** Xiaoshuang Wei, Zesheng Wei, Mingchong Yang, Qiang Chen, Jingzhen Wang, Liyan Zhao, Xia Zhang, Lingqiang Wang, Lihua Hu

**Affiliations:** 1 State Key Laboratory of Conservation and Utilization of Subtropical Agricultural Biological Resources, Guangxi University, Nanning, China; 2 Guangxi Key Laboratory of Sugarcane Biology, College of Agriculture, Guangxi University, Nanning, China; 3 College of Plant Science and Technology, Huazhong Agricultural University, Wuhan, China

**Keywords:** sugarcane, cell wall, cellulose synthase, gene family, phylogenetic analysis, stem

## Abstract

Sugarcane is the most critical sugar and bioenergy crop. Cellulose synthase (*CESA*) genes are involved in cellulose synthesis, playing a significant role in plant growth and development, as well as in secondary metabolism. In this study, we systematically analyzed the *CESA* gene family in Sugarcane (*Saccharum spontaneum*), recognizing 30 members. 32 pairs of segmental duplication events contributed mainly to the expansion of this superfamily. In addition to plant hormones, stress-responsive and growth-related cis-acting elements were identified in *SsCESA* gene promoters, along with many MYB, NAC, and WRKY transcription factor binding sites. Transcriptomic data showed that most SsCESA transcripts were higher in stems and leaf transitional parts. qRT-PCR assays confirmed that *SsCESA* genes could respond to cold, heat, and salt stress. GFP subcellular localization assays exhibited that SsCESA4 and SsCESA9 proteins were localized at the Golgi apparatus. Finally, EMSA experiments confirmed that SsNST1 could bind to the promoters of *SsCESA4*, *SsCESA7*, and *SsCESA9*. This research elucidates the evolutionary patterns and functional characteristics of the SsCESA gene family in sugarcane, providing a basis for future investigations into its mechanism of action.

## 1 Introduction

Sugarcane is the most crucial sugar and bioenergy crop, accounting for 80% of global sugar production and 40% of ethanol production (Grivet and Arruda, 2002; Lam et al., 2009). There is increasing focus on using sugarcane biomass for second-generation biofuels from lignocellulosic materials (Hans et al., 2023). Sugarcane achieves the highest dry biomass yield, amounting to nearly 39 tons per hectare annually, surpassing other crops such as miscanthus, corn, and switchgrass (Kandel et al., 2018; Ain et al., 2022). A comprehensive analysis of the genetic basis of the components that comprise sugarcane biomass and stalk lodging resistance is essential to increase yield (Li et al., 2023).

The growth, biomass formation, and mechanical strength of sugarcane stalks are closely linked to the regulation of intrinsic cell wall biosynthesis. In addition, the cell wall serves as the primary defense in plants, acting as a barrier against environmental changes (Son et al., 2025). It consists of the primary and secondary walls. The primary wall is a thin layer formed during cell division and elongation, while the secondary wall is thicker and serves specific cell types with specialized functions. Cellulose, hemicellulose, and lignin constitute the major components of the secondary cell wall (Zhong and Ye, 2015). Cellulose is a strong, effectively irreversible C-sink in plants, and increasing cellulose in stalk walls could improve stalk strength and harvest index (Appenzeller et al., 2004; Salimi et al., 2024). During sugarcane culm maturation, cellulose synthesis is regulated by the coordinated expression of genes, including those encoding cellulose synthases and cellulose synthase-like proteins (Casu et al., 2007; Casu et al., 2015). Also, there is a strong association between CESA expression and cell wall metabolism, suggesting a shift in the timing of carbon partitioning in the apical culms of sugarcane (Hosaka et al., 2021). These genes are closely related to plant height, tillering, and stress resistance.

Cellulose is synthesized at the plasma membrane. The biosynthesis of cellulose comprises three key stages: initiation of the beta-1,4-glucan chain, elongation of the chain, and assembly of microfibrils (Turner and Kumar, 2017). Many genes have been characterized that contribute to the formation of cellulose synthase complexes (CSCs) (Li et al., 2014; Polko and Kieber, 2019). The cellulose synthase gene (*CESA*) family, part of the cellulose synthase-like superfamily, plays a key role in cellulose biosynthesis. *CESA* genes in higher plants were initially identified in elongating cotton (*Gossypium hirsutum*) fibers (Pear et al., 1996). Subsequently, the gene family discovered in cotton and rice (*Oryza sativa*) was designated as *CESA* (Little et al., 2018). The CESA protein sequence is highly conserved and contains GT2 and ZF domains, with eight transmembrane domains distributed into two regions: two spanning the N-terminal region and six spanning the C-terminal region (Kurek et al., 2002; Kaur et al., 2016). CSC is a rosette-like structure and consists of six globular protein complexes, with each complex containing several structurally similar CESA subunits. *AtCESA1*, *AtCESA3*, and *AtCESA6* in *Arabidopsis* and *OsCESA1*, *OsCESA3*, and *OsCESA8* in rice which have been identified for primary cell wall (PCW) formation, whereas *AtCESA4*, *AtCESA7*, and *AtCESA8* and *OsCESA4*, *OsCESA7*, and *OsCESA9* are responsible for cellulose biosynthesis in the secondary cell walls (SCW), respectively (Tanaka et al., 2003; Taylor et al., 2003; Persson et al., 2007; Wang et al., 2010).

*CESA* genes play significant roles in plant growth and development and in secondary metabolism. Decreased cellulose production results in lignin formation, which is partially regulated by jasmonic acid, ethylene, and additional signaling pathways (Mielke and Gasperini, 2019; Zhou et al., 2024). Numerous *CESA* mutants exhibit severe defects in cellulose synthesis. In *Populus trichocarpa*, the complete loss of *PtrCesA4*, *7A/B*, or *8A/B* results in a 90% decrease in cellulose content (Xu et al., 2021). Also, mutations in *CESA4* cause a 37–67% reduction in cellulose content in poplar (Nayeri et al., 2022). The cellulose content in culms of *OsCESA4*, *OsCESA7*, and *OsCESA9* null mutants was significantly reduced (8.9%–25.5% of wild-type) (Tanaka et al., 2003). Nevertheless, overexpression of three PCW *AtCESA6*-like genes (*AtCESA2*, *AtCESA5*, and *AtCESA6*) significantly promotes plant growth and increases biomass accumulation by enhancing cell expansion and thickening cell walls in transgenic *Arabidopsis* (Hu et al., 2018). When plants are under stress, cellulose undergoes remodeling by altering its biosynthesis processes, which involves modulating the activities of CSCs and *CESA* gene expression (Zhang et al., 2025a). Disruption of *AtCesA8/IRX1* enhances tolerance to drought and osmotic stress (Chen et al., 2005). Mutants of *AtCESA8* and *AtCESA3* exhibit increased resistance to salt stress. The *OsCESA9*/*OsCESA9^D387N^* heterozygous plants increase salt tolerance by scavenging and detoxifying ROS, and this effect is indirectly reflected in the expression of related genes (Ye et al., 2021). The expression of *CESA* genes is precisely controlled by transcription factor networks. In banana, MaNAC1 activates MaCESA7 and MaCESA6B, thus increasing cellulose at low temperature (Yin et al., 2024). In cotton, GhFSN subclade (GhFSN1A and GhVNI1A) activates the *GhCESA* (Chen et al., 2025).

Some studies focus on the *CESA* gene family involved in cellulose production in crops and trees, covering gene identification, activity, and function (Guo et al., 2021; Zhang et al., 2021a; Sod et al., 2023; Wang et al., 2023). However, there are no reports on this gene family in sugarcane, and the regulatory network underlying cell wall biosynthesis is also poorly understood. Studies of *CESA* genes provide a foundation for uncovering mechanisms of plant cell wall biosynthesis, as *CESAs* can serve as marker genes to identify new genes involved (Polko and Kieber, 2019). Using marker genes, co-expression analysis, EMSA, and Y1H can reveal roles of transcription factors in regulating genes and in the biosynthesis of plant cell walls (Ruprecht and Persson, 2012; Taylor-Teeples et al., 2015; Zhang et al., 2025b).To investigate the potential role of sugarcane *CESA* genes in growth, development, and stress responses, we identified and analyzed all *CESA* genes in the sugarcane (*S. spontaneum*) (AP85-441) genome. The sequence, structure, evolution, and expression pattern of these genes were systematically studied. The subcellular localization of SsCESA4 and SsCESA9 proteins further strengthened the function of the *CESAs* in cellulose biosynthesis. Additionally, the transcription factor SsNST1 was shown to bind the promoters of *SsCESA4*, *SsCESA7*, and *SsCESA9*. This study classifies the *CESA* genes in sugarcane growth, development, and stress response, offering potential for genetic improvement.

## 2 Result

### 2.1 Identification and distribution of *SsCESA* genes in the *S. spontaneum* genome

**Figure 1:**
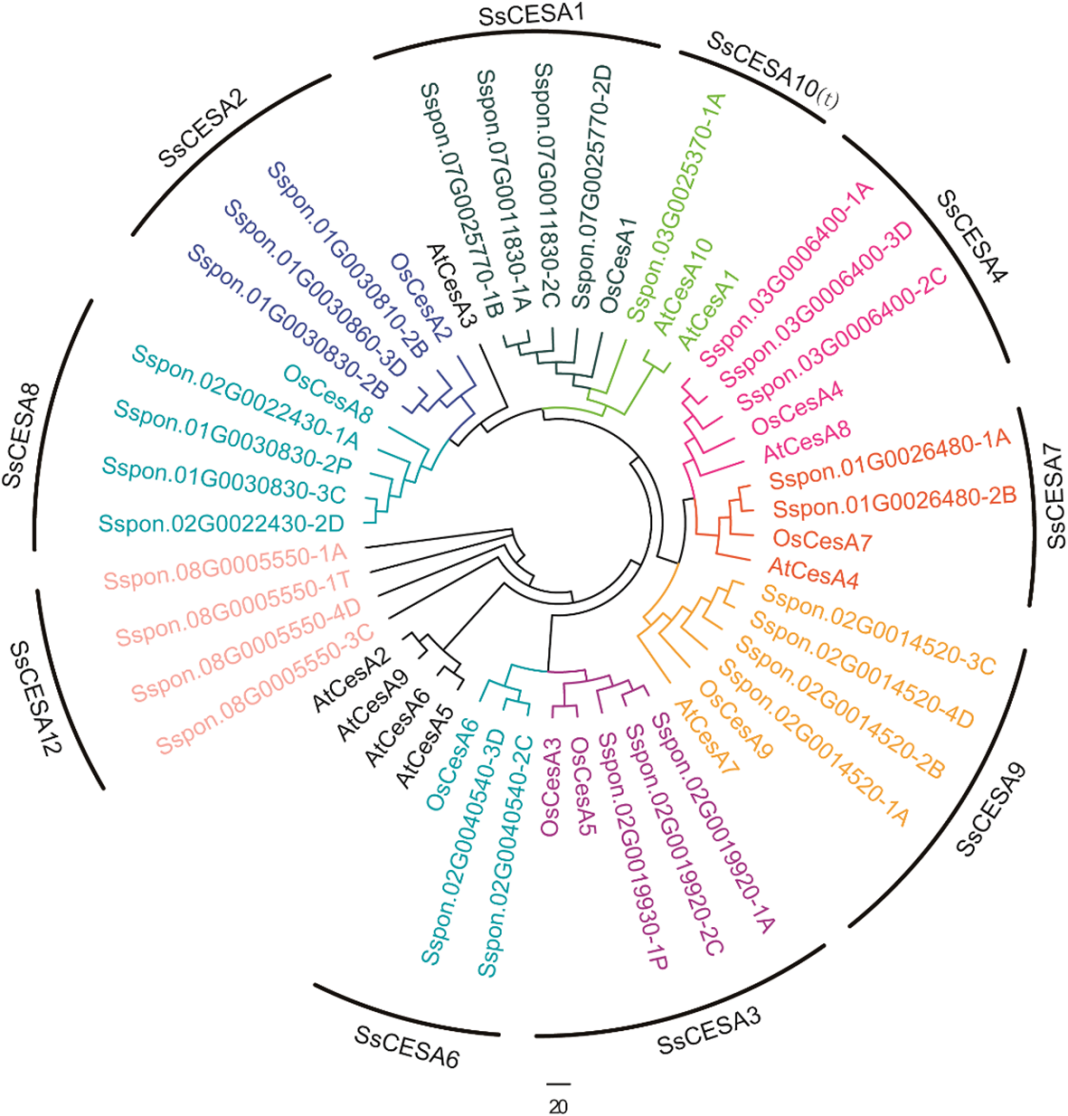
The phylogenetic relationships among CESA proteins of sugarcane, rice, and *Arabidopsis*.

A total of 30 CESA family genes were found in the sugarcane *S.* *spontaneum* genome. The *SsCESA* gene was named based on its homolog in rice, and each *CESA* gene in sugarcane has one to four copies. Therefore, each gene copy is identified by a number and a letter following the gene name (**Figure 1** & **Table 1**). Sspon.03G0006400, Sspon.01G0026480, and Sspon.02G0014520 can be clustered with OsCESA4, OsCESA7, OsCESA9, and their *Arabidopsis* counterparts, AtCESA4, AtCESA7, and AtCESA8, related to secondary cell walls. These were named SsCESA4, SsCESA7, and SsCESA9. Sspon.02G0019920-1A, Sspon.02G0019920-2C and Sspon.02G0019930-1P clustered in the same clade as OsCESA3 and OsCESA5 of rice (**Figure 1**). Thus, we designated them as SsCESA3.1-1A, SsCESA3.1-2C, and SsCESA3.2-1P, respectively. Moreover, there is no *SsCESA5* in *S. spontaneum*. Sspon.08G0005550-1A/3C/4D/1T did not cluster with any CESA members of rice or *Arabidopsis* (**Figure 1**). Furthermore, OsCESA10 &CESA11 were found to contain only a CS domain, and CESA10 clustered with CESA-like proteins in rice (OsCSLs) (Wang et al., 2010). Therefore, *Sspon.08G0005550* was designated as SsCESA12.Sspon.03G0025370-1A clusters with AtCESA1 and AtCESA10. AtCESA1, AtCESA3, and AtCESA6 in *Arabidopsis*, and OsCESA1, OsCESA3, and OsCESA8 in rice, are associated with the primary cell wall. Sspon.07G0011830-1A/2C and Sspon.07G0025770-1B/2D have higher homology with OsCESA1, so they are respectively named SsCESA1.1-1A/2C and SsCESA1.2-1B/2D. *Sspon.03G0025370-1A* does not belong to CESA1 members, but it is close to AtCESA10, so it was temporarily named SsCESA10-1A(t), with “t” being the abbreviation of “temporarily”. Except for the relatively short amino acid sequences of SsCESA8.1-3C, SsCESA9-2B, SsCESA9-4D, and SsCESA12-1T, the amino acid sequences of the remaining SsCESA are approximately 1000 amino acids in length (**Table 1**). All SsCESA proteins were predicted to be hydrophilic proteins (**Table 1**). To predict the subcellular localization of the SsCESA protein, we utilized WoLF PSORT and LocTree3 (Goldberg et al., 2014) (**Supplementary Table 1**). The WoLF PSORT indicated that nearly all proteins are localized to plastids. The LocTree3 indicated that the SsCESA proteins are more likely to be located in the cell membrane, endoplasmic reticulum, and Golgi apparatus. Furthermore, the possibility of SsCESA1, SsCESA3, SsCESA8, SsCESA4, and SsCESA10(t) proteins being located in the Golgi apparatus is higher than in the cell membrane.

**Table 1:** Basic information of *SsCESA*.

Number	Gene	Gene name	Number of amino acids	Molecular weight	Theoretical pI	Instability index	Aliphatic index	Grand average of hydropathicity
1	*Sspon.01G0026480-1A*	*SsCESA7-1A*	1105	124371.96	7.59	37.54	79.21	-0.246
2	*Sspon.01G0026480-2B*	*SsCESA7-2B*	1087	122156.4	7.17	36.71	79.9	-0.228
3	*Sspon.01G0030810-2B*	*SsCESA2.1-1A*	1012	114282.26	8.13	36.98	84.08	-0.207
4	*Sspon.01G0030830-2B*	*SsCESA2.2-2B*	988	111276.8	8.28	38.55	82.39	-0.201
5	*Sspon.01G0030860-3D*	*SsCESA2.3-3D*	1060	119503.09	8.13	38.53	82.93	-0.23
6	*Sspon.01G0030830-2P*	*SsCESA8.1-2P*	1081	120930.49	7.69	37.89	81.52	-0.221
7	*Sspon.01G0030830-3C*	*SsCESA8.1-3C*	772	86056.37	6.7	40.51	69.44	-0.535
8	*Sspon.02G0014520-1A*	*SsCESA9-1A*	1051	118171.05	6.36	35.81	84.58	-0.16
9	*Sspon.02G0014520-2B*	*SsCESA9-2B*	735	82513.29	6.01	40.01	73.71	-0.408
10	*Sspon.02G0014520-3C*	*SsCESA9-3C*	1054	118718.86	6.44	35.68	85.45	-0.141
11	*Sspon.02G0014520-4D*	*SsCESA9-4D*	415	46454.69	4.85	35.31	82.41	-0.366
12	*Sspon.02G0019920-1A*	*SsCESA3.1-1A*	1152	130376.92	6.66	39.77	82.66	-0.182
13	*Sspon.02G0019920-2C*	*SsCESA3.1-2C*	1039	117523.55	6.53	38.87	78.98	-0.308
14	*Sspon.02G0019930-1P*	*SsCESA3.2-1P*	1030	115608.14	6.29	41.15	70.35	-0.498
15	*Sspon.02G0022430-1A*	*SsCESA8.2-1A*	1081	121072.56	7.91	37.4	81.25	-0.235
16	*Sspon.02G0022430-2D*	*SsCESA8.2-2D*	1106	124239.26	8.33	38	80.13	-0.273
17	*Sspon.02G0040540-2C*	*SsCESA6-2C*	1094	122487.86	7.42	44.64	82.36	-0.19
18	*Sspon.02G0040540-3D*	*SsCESA6-3D*	1138	127527.33	7.04	44.95	81.14	-0.198
19	*Sspon.03G0006400-1A*	*SsCESA4-1A*	977	109781.15	6.25	39.84	83.95	-0.109
20	*Sspon.03G0006400-2C*	*SsCESA4-2C*	981	110324.68	6.04	40.6	83.9	-0.118
21	*Sspon.03G0006400-3D*	*SsCESA4-3D*	977	109781.15	6.25	39.84	83.95	-0.109
22	*Sspon.03G0025370-1A*	*SsCESA10 -1A(t)*	1062	119140.63	5.99	36.21	89.3	-0.128
23	*Sspon.07G0011830-1A*	*SsCESA1.1-1A*	1072	120872.71	6.6	39.97	84.75	-0.204
24	*Sspon.07G0011830-2C*	*SsCESA1.1-2C*	1073	120919.72	6.6	40.05	84.67	-0.203
25	*Sspon.07G0025770-1B*	*SsCESA1.2-1B*	1060	119754.89	8.03	37.71	85.96	-0.168
26	*Sspon.07G0025770-2D*	*SsCESA1.2-2D*	1073	120913.76	6.6	39.97	84.76	-0.201
27	*Sspon.08G0005550-1A*	*SsCESA12-1A*	1066	121110.49	8.16	38.66	86.57	-0.183
28	*Sspon.08G0005550-1T*	*SsCESA12-1T*	677	77387.88	7.32	41.93	76.29	-0.478
29	*Sspon.08G0005550-3C*	*SsCESA12-3C*	978	110434.8	7.29	38.58	82.38	-0.223
30	*Sspon.08G0005550-4D*	*SsCESA12-4D*	1071	121632.1	8.22	39.13	85.53	-0.192

**Figure 2:**
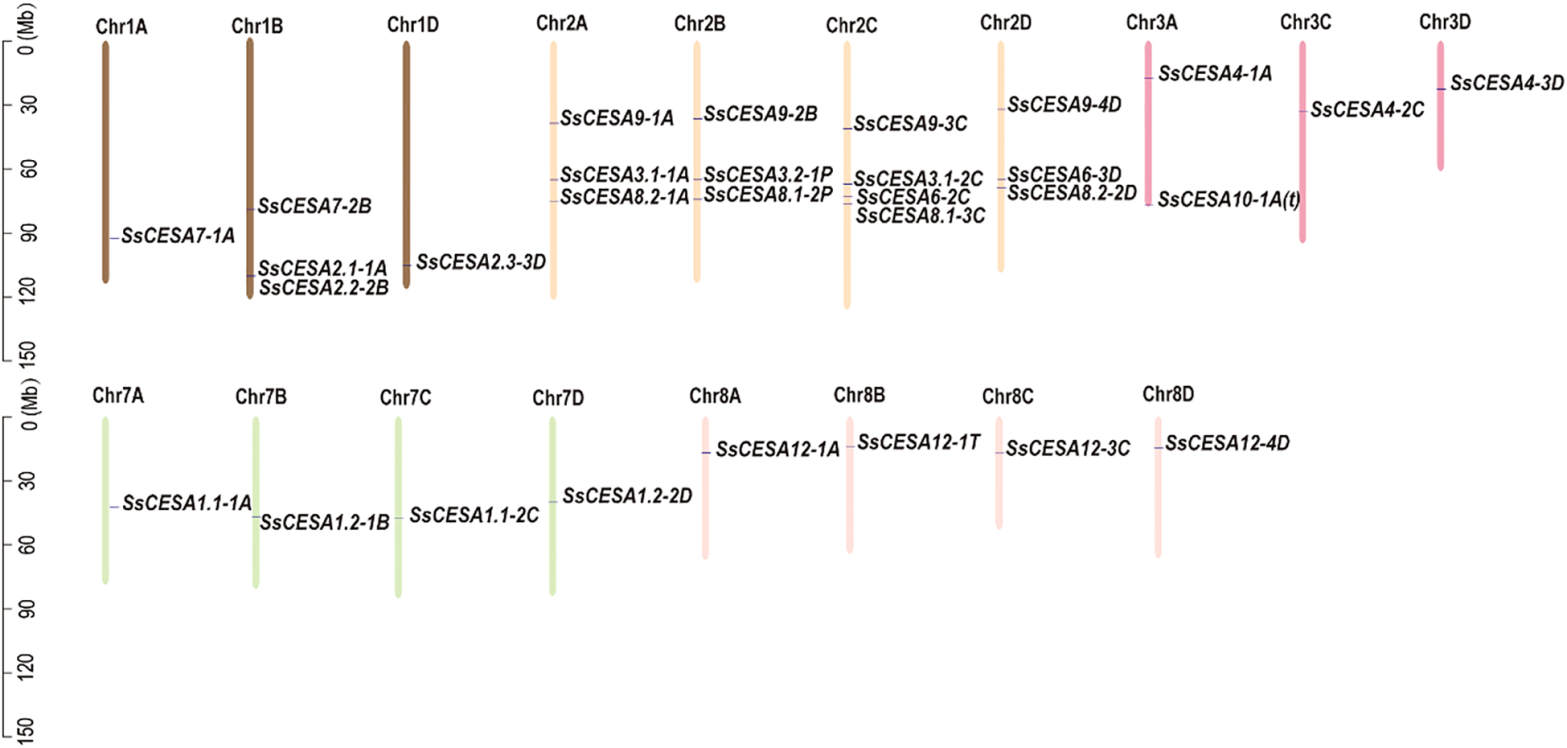
Distribution of the *SsCESA* gene family members on *S. spontaneum* chromosomes.

The 30 *SsCESA* genes were unevenly distributed across 18 chromosomes (**Figure 2**). These genes are found only on chromosomes Chr1, Chr2, Chr3, Chr7, and Chr8, with the highest number on Chr2, which has 13 genes.

### 2.2 Motifs, conserved domains, and gene structure of the *SsCESA* gene family

**Figure 3:**
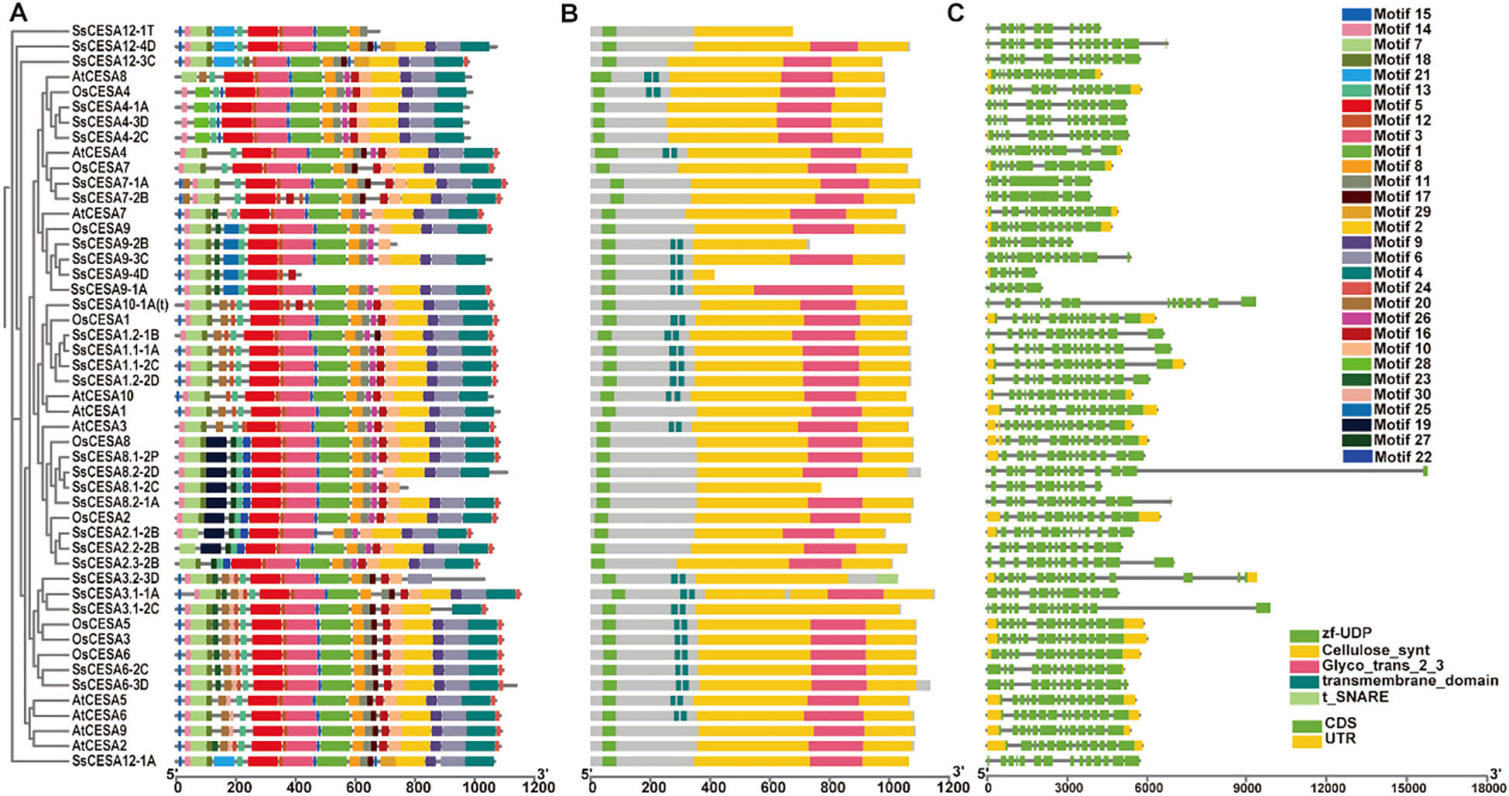
Motifs, conserved domains, and gene structure of the CESA gene family among *S. spontaneum*, rice, and *Arabidopsis*. **(A)** Analysis of conserved motifs of SsCESA, OsCESA, and AtCESA proteins. 30 conserved protein motifs are exhibited in different colors. **(B)** Domain analysis in SsCESA, OsCESA, and AtCESA proteins. **(C)** Gene-structure of the *SsCESA*, *OsCESA*, and *AtCESA* gene family, CDS depicted by green, UTR by yellow, whereas straight lines represent introns.

Analysis of conserved protein motifs shows the SsCESA gene family comprises 30 motifs. All SsCESA proteins include motifs 12 and 13; most also contain motifs 1, 3, 5, 8, and 11. (**Figure 3A**). Additionally, specific motifs are gene-specific, such as motif 21 in SsCESA12, motif 28 in SsCESA4, and motif 25 in SsCESA9 (**Figure 3A**).

Five conserved domains were also detected in the SsCESA, OsCESA, and AtCESA proteins (**Figure 3B**). All the CESA proteins contained zf-UDP and Cellulose_synt domain. Transmembrane domains are exclusively detected in SsCESA1, SsCESA3, SsCESA6, and SsCESA9 proteins (**Figure 3B**). All CESA proteins, except for SsCESA12-1T, SsCESA9-2B/4D, SsCESA8.1-2C, SsCESA3.2-3D, and SsCESA3.1-2C, contain a complete GT2 domain.

The *SsCESA* genes have a relatively high number of exons, ranging from 5 to 14 (**Figure 3C**). *SsCESA1*, *SsCESA3*, *SsCESA4*, *SsCESA6*, and *SsCESA8* show a high degree of similarity in gene structure to the rice and *Arabidopsis* genes within their respective clades. All those results showed that partial CESA family members are evolutionarily conserved in rice, *Arabidopsis thaliana*, and *S. spontaneum*.

### 2.3 Evolutionary and collinearity analysis of *SsCESA* genes

**Figure 4:**
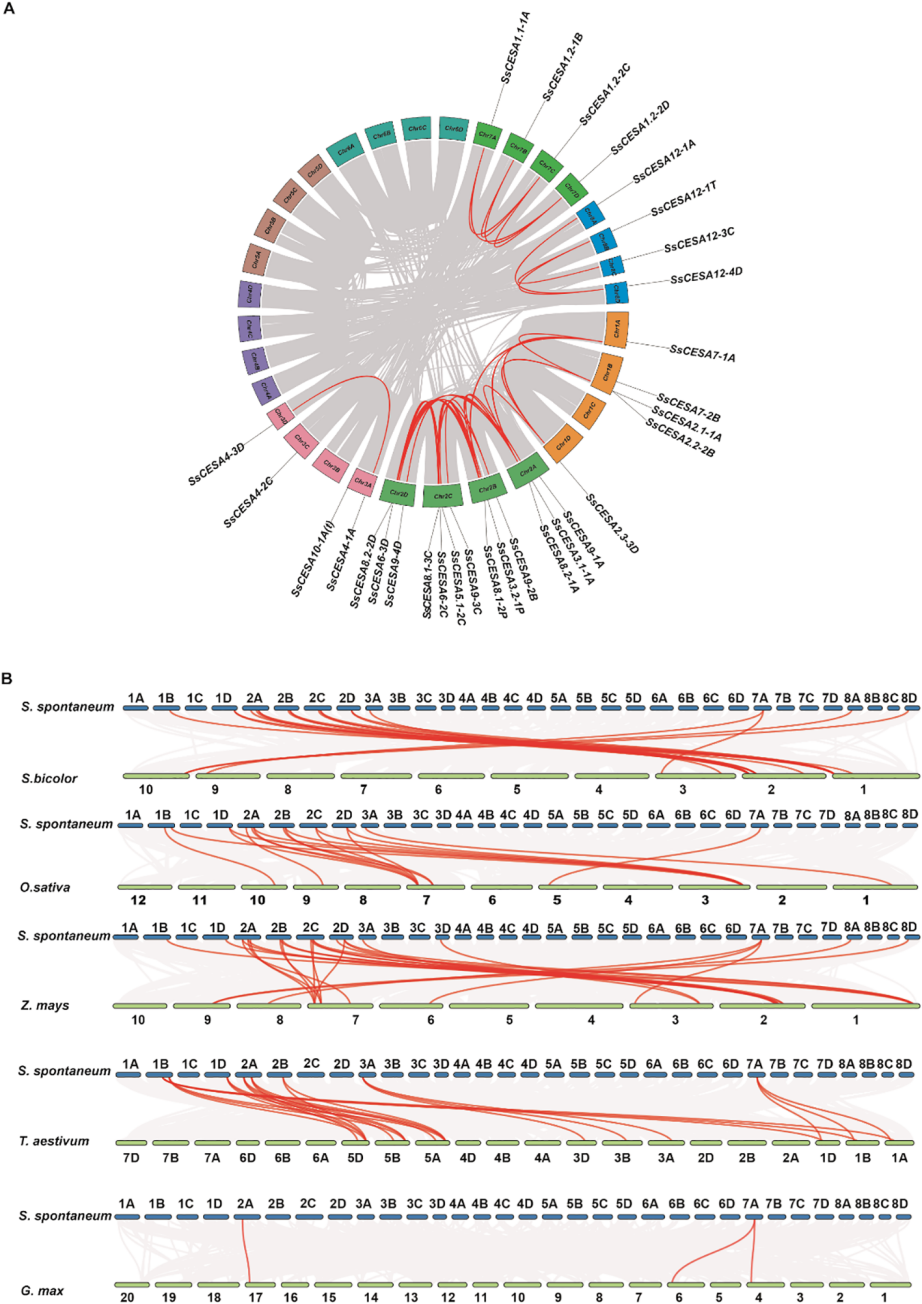
Collinear relationships of the *SsCESAs* within its genome and with *Sorghum bicolor*, *Oryza sativa*, *Zea mays, Triticum aestivum*, and *Glycine max.***(A)** Collinear Analysis within the *SsCESA* Gene Family. The red lines represent the collinear associations among *SsCESA* genes, while the gray lines depict the collinear relationships across all genes. **(B)** Collinearity analysis of *SsCESAs* genes in comparison with *Sorghum bicolor*, *Oryza sativa*, *Zea mays, Triticum aestivum*, and *Glycine max.* The red lines represent collinear associations among *SsCESA* genes across species, while the gray lines depict overall collinear gene relationships between the two species.

The collinearity analysis revealed 32 pairs of segmental duplication events involving 30 *SsCESA* genes in *S. spontaneum*. Multiple genes were identified for *SsCESA1*, *SsCESA2*, *SsCESA3*, and *SsCESA8*, collinearity was also detected between *SsCESA1.1* and *SsCESA1.2*, *SsCESA2.1* and *SsCESA2.1*, *SsCESA3.1* and *SsCESA3.2*, as well as *SsCESA8.1* and *SsCESA8.2* (**Figure 4A**; **Supplementary Table 2**). This finding suggests that the members of the *SsCESA* gene family were amplified via the segmental duplication.

To better understand the evolutionary relationships of the *SsCESA* gene family across species, we performed collinearity analysis with the CESA proteins from *S. spontaneum*, four monocotyledonous plants (*Sorghum bicolor*, *Oryza sativa*, *Zea mays*, and *Triticum aestivum*), and a dicotyledonous plant (*Glycine max*). Syntenic analyses were performed across these genomes and showed that there were 20 collinear events between *S. spontaneum* and sorghum, 17 with rice, 27 with maize, 23 with wheat, and only 3 with soybean (**Figure 4B**; **Supplementary Tables 3–7**). Collinearity events were detected for SsCESA1, SsCESA2, SsCESA4, SsCESA7, SsCESA8, and SsCESA9 across the four monocotyledonous species. These results indicate that the *SsCESA* gene family is relatively conserved and that *SsCESA* genes are more closely related to those in *Z*. *mays* than to those in *Sorghum bicolor.*

### 2.4 Predicting *cis*-acting regulatory elements in the promoter regions of the *SsCESA* gene family

**Figure 5:**
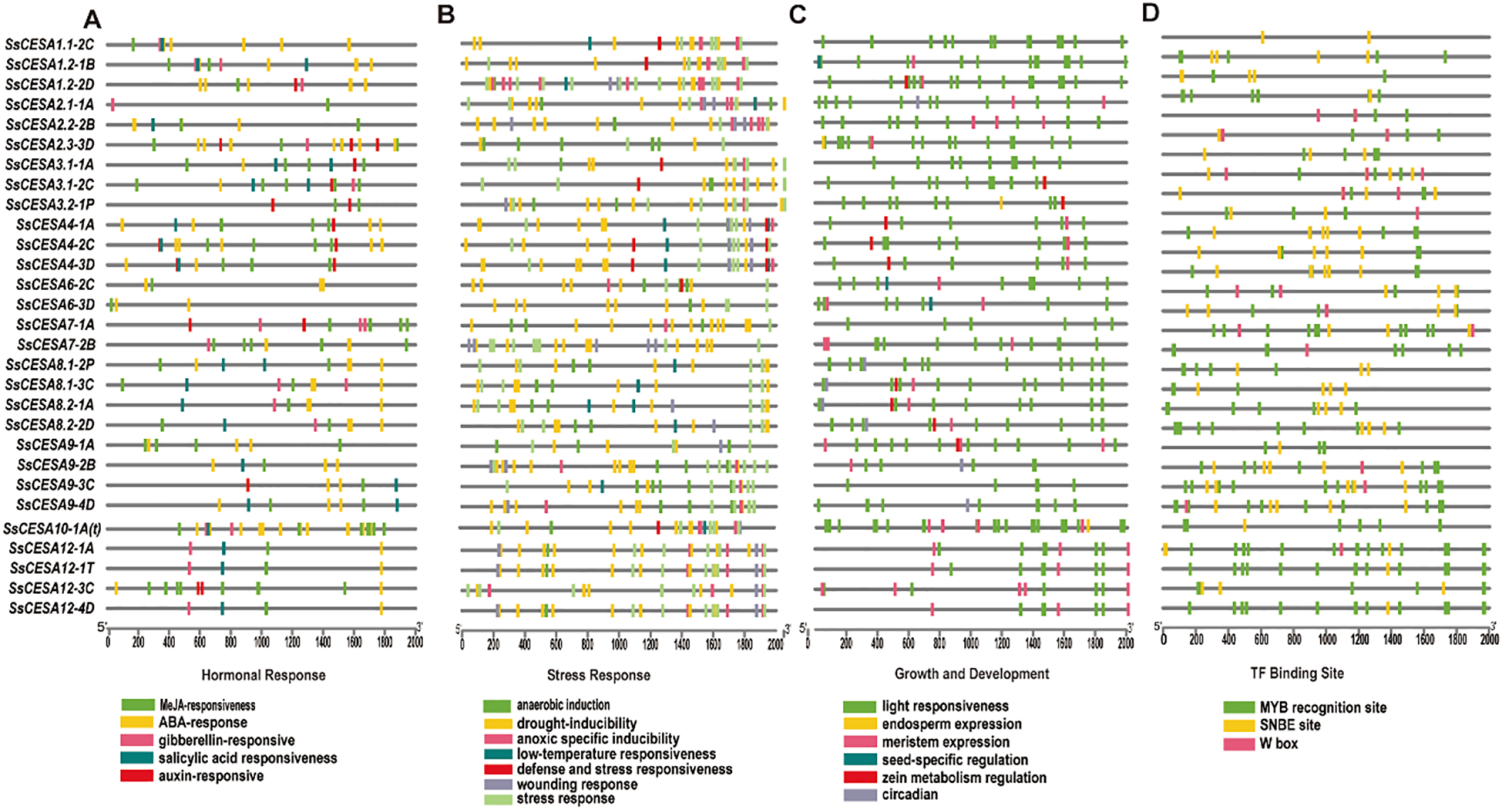
*Cis*-acting regulatory element (CRE) distribution on the predicted promoter regions of *SsCESA* genes. **(A)** Hormonal responsiveness. **(B)** Stress responsiveness. **(C)** Growth and development. **(D)** TF binding site.

*Cis*-acting regulatory elements serve as crucial molecular regulators, playing a key role in transcriptional control by modulating a complex, dynamic network of gene expression. This network governs a wide range of biological functions, such as responses to abiotic stress, hormonal signaling, and developmental mechanisms (Yamaguchi-Shinozaki and Shinozaki, 2005). Four hormone-response elements, namely MeJA-responsiveness, ABA-responsiveness, auxin-responsiveness, and gibberellin-responsiveness, were investigated in this study. Among them, the largest number of *cis*-acting elements is associated with MeJA-responsiveness, totaling 147 (**Figure 5A**). For stress responses, the number of *cis*-acting elements related to drought induction is the largest, reaching 199 (**Figure 5B**). These results suggest that *SsCESA* plays a crucial role in response to drought stress. Among the classifications of cis-acting elements associated with growth and development, those related to light response exhibit the highest proportion (**Figure 5C**). The NAC, MYB, and WRKY transcription factor families are prominent. They regulate downstream genes via SNBE sites, MYB binding sites, and W–box elements, respectively (Dubos et al., 2010; Rushton et al., 2012; Nuruzzaman et al., 2013). When predicting the cis-acting elements in the promoter region of *SsCESA*, we found that, except for *SsCESA1.1-2C*, the promoters of the other genes have at least two MYB binding sites (**Figure 5D**). In addition to *SsCESA2.2-2B*, the promoter regions of all other genes contain SNBE sites. We have also identified W-box elements in the promoter regions of *SsCESA2*, *SsCESA3*, *SsCESA6*, *SsCESA7*, *SsCESA9*, and *SsCESA12*. These results suggest that NAC, MYB, and WRKY transcription factors may regulate *SsCESA*.

### 2.5 *SsCESA* gene expression in growth and development

**Figure 6:**
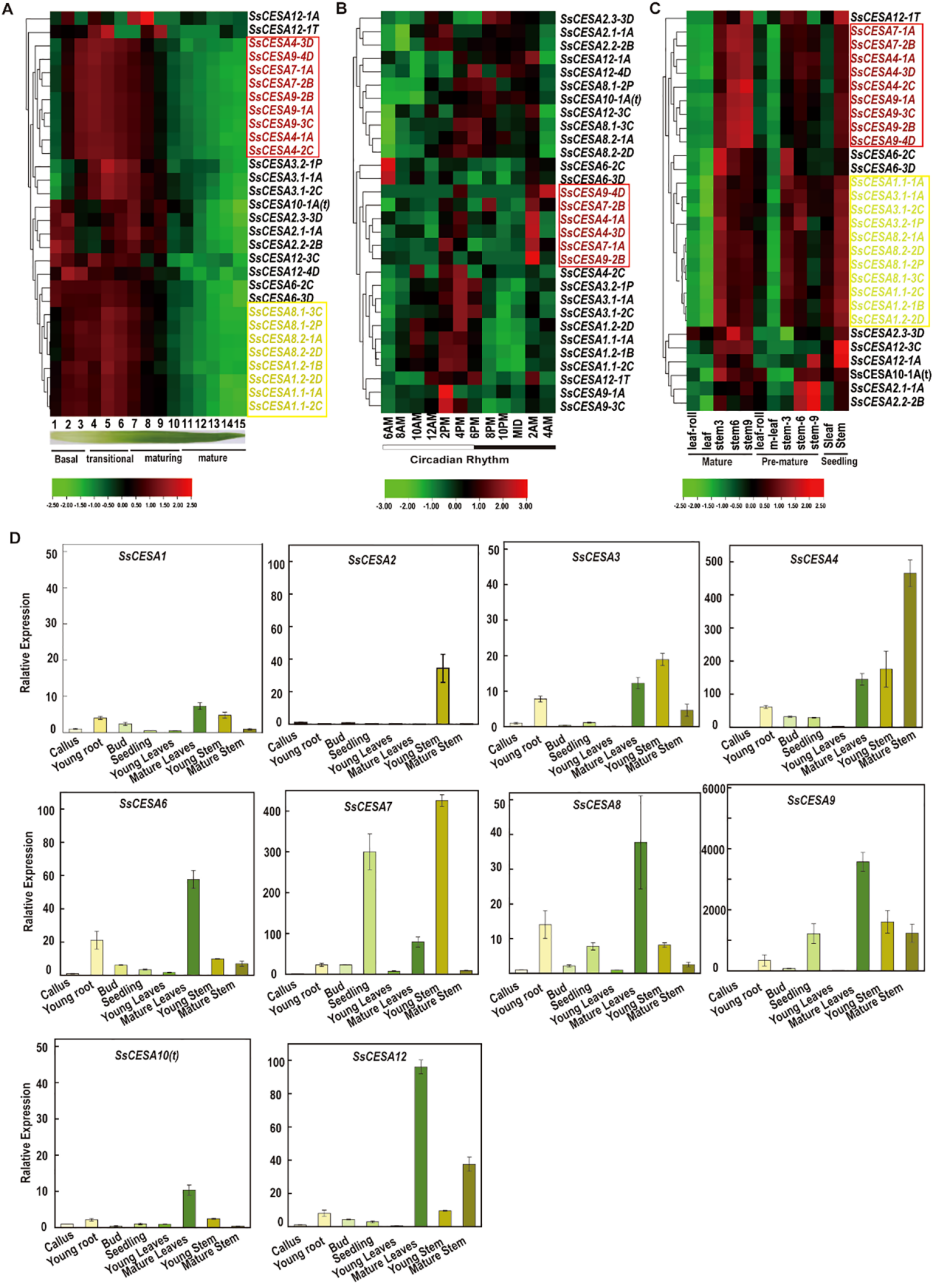
SsCESA gene expression pattern **(A)** Leaf developmental gradient. **(B)** Circadian rhythms. **(C)** Tissues at different developmental stages. The color scale on the right side of each heatmap represents relative gene expression levels, with red indicating higher expression, green indicating lower expression, and black indicating intermediate expression. **(D)** The expression levels of the *SsCESA* gene across various tissues were analyzed using quantitative real-time PCR (qRT-PCR).

We utilized three publicly available transcriptomic datasets of *S.* *spontaneum* to investigate the expression pattern of the *SsCESA* gene. Almost all the *SsCESA* genes exhibited their highest expression levels during the transitional phase of leaf development (**Figure 6A**; **Supplementary Table 9**).

Except for S*sCESA2.1-1A* and *SsCESA2.2-2B*, which exhibit relatively high expression levels in the circadian rhythm expression profiles, these genes might play crucial roles in regulating the circadian rhythm (**Figure 6B**; **Supplementary Table 8**). The expression levels of the remaining *SsCESA* genes are relatively low. Among them, the FPKM (Fragments Per Kilobase of exon per Million reads mapped) values of *SsCESA9-1A/2B/3C/4D* are close to zero (**Figure 6B**; **Supplementary Table 8**), and the *SsCESA4*, *SsCESA7*, and *SsCESA9* also cluster together. The *CESA* gene family that regulates cellulose formation has been found to exhibit predominant expression in the stems, particularly within the xylem tissue, across various plant species, such as *Triticum aestivum* (Kaur et al., 2016), *Miscanthus* × *giganteus* (Zeng et al., 2020), and *Linum usitatissimum* (Mokshina et al., 2014). We further observed that the *SsCESA* genes demonstrated relatively high expression levels in the stem across all stages. Notably, their expression peaked in mature stems (**Figure 6C**; **Supplementary Table 9**). In addition, *SsCESA4*, *SsCESA7*, and *SsCESA9* clustered together, while *SsCESA1*, *SsCESA3*, and *SsCESA8* formed another cluster. All of these results suggest that *SsCESA4*, *SsCESA7*, and *SsCESA9* in *S. spontaneum* exhibit similar spatio-temporal expression patterns.

To confirm the expression patterns of *SsCESA* across growth and development, we collected eight tissue types at various stages. We conducted qRT-PCR tests to verify the expression of the *SsCESA* gene family. *SsCESA4/7/9* was expressed at higher levels in the young stems and mature stems, consistent with the transcriptional profiles associated with growth and developmental processes (**Figures 6C, D**). The expression levels of *SsCESA1*, *SsCESA3*, *SsCESA6*, *SsCESA8*, and *SsCESA12* are relatively high in mature leaves.

SsCESA1, SsCESA3, and SsCESA8 proteins display relatively high homology with the CESA proteins related to the primary cell walls of rice (OsCESA1, OsCESA3, and OsCESA8) and *Arabidopsis thaliana* (AtCESA1, AtCESA3, and AtCESA6) (**Figure 1**). Additionally, co-expression analysis indicates that *SsCESA1*, *SsCESA3*, and *SsCESA8* share similar expression patterns. Higher expression of *SsCESA1*, *SsCESA3*, and *SsCESA8* occurs in rapidly elongating tissues like the basal parts of leaves, seedling leaves, and young stems, indicating their role in primary cell wall biosynthesis, as supported by their high homology with primary cell wall-related *CESA*s in rice and Arabidopsis. *SsCESA4*, *SsCESA7*, and *SsCESA9* are more expressed in maturing stems, aligning with their role in secondary cell wall thickening. However, their low expression in young internodes (PCW-dominant stage) suggests dynamic regulation during sugarcane stem maturation. Arabidopsis mutants of *AtCESA4/7/8* (SCW *CESAs*) exhibit collapsed xylem vessels (Turner and Somerville, 1997), highlighting the conservation of SCW-related CESA function in dicots and monocots. Combined with the co-expression analysis of the three transcriptome databases, it was found that *SsCESA4*, *SsCESA7*, and *SsCESA9* cluster together and have relatively high expression levels in the stem. Therefore, we speculate that *SsCESA4*, *SsCESA7*, and *SsCESA9* are associated with the secondary cell wall. These results suggest that *CESA* genes play a crucial role in the development of stems. Moreover, *CESA* genes with similar functions exhibit the same co-expression pattern.

### 2.6 *SsCESA* gene expression in stress responses

**Figure 7:**
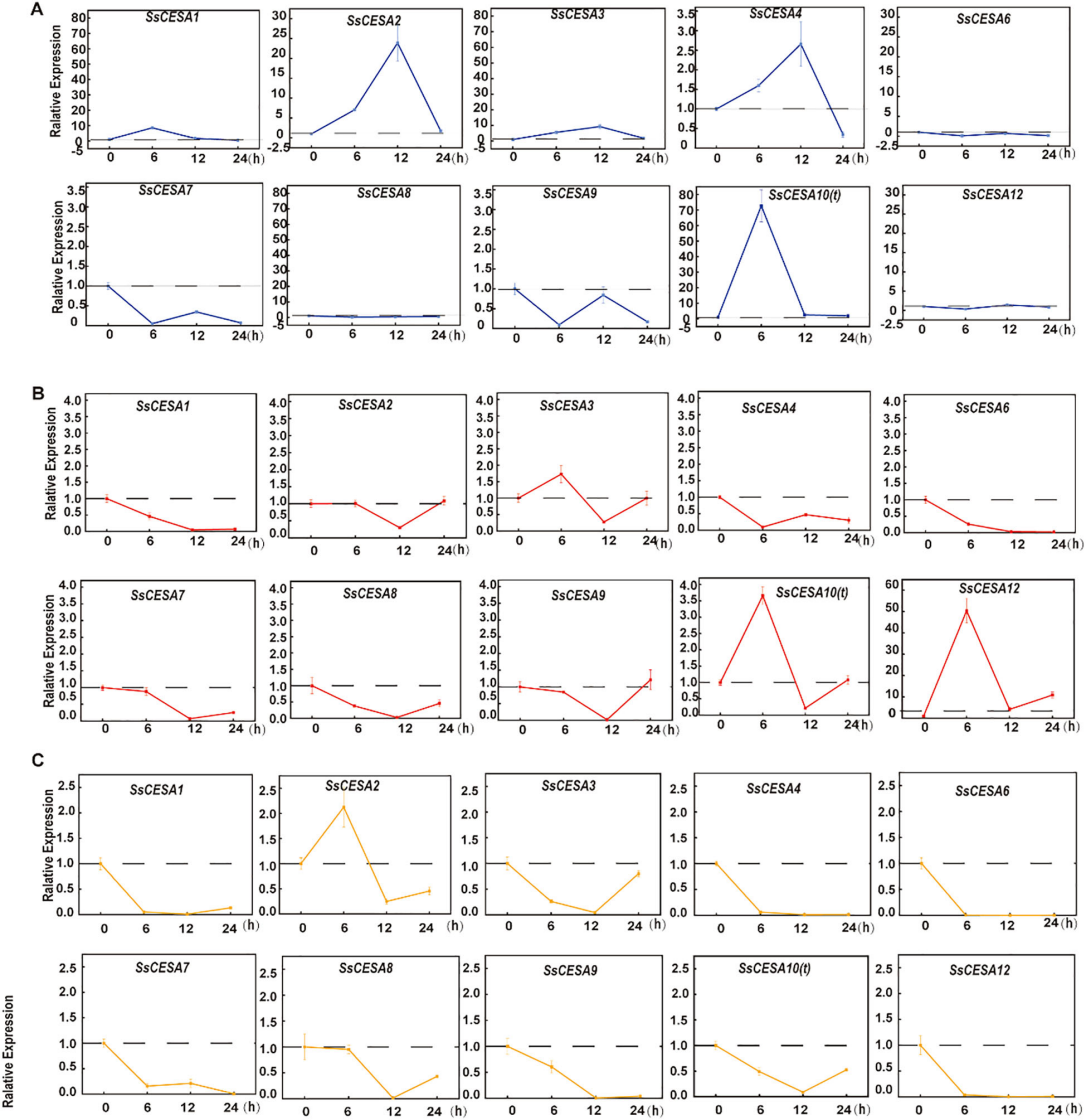
Analysis of the expression patterns of *SsCESA* genes using qRT-PCR at time points 0, 6, 12, and 24 hours under **(A)** Cold stress. **(B)** Heat stress. **(C)** Salt stress.

The cell wall serves as the primary defense mechanism in plants, acting as a protective barrier against external environmental fluctuations (Son et al., 2025). Cellulose synthase (CESA) is the core enzyme responsible for cellulose biosynthesis in plants, and its activity directly determines the mechanical strength and function of the cell wall (Richmond, 2000). We examined the expression levels of *SsCESA* genes under cold (6°C), heat (45°C), and salt stress (150 mmol/L) conditions. Following cold treatment, the expression levels of *SsCESA1*, *SsCESA2*, *SsCESA3*, *SsCESA4, and SsCESA10(t)* were quickly induced. Specifically, the expression levels of *SsCESA1.1* and *SsCESA10(t)* peaked at 6 hours and then decreased by 12 hours. Conversely, the expression levels of *SsCESA3* and *SsCESA4* peaked at 12 hours and returned to baseline by 24 hours (**Figure 7A**). *SsCESA6, SsCESA7, SsCESA8, SsCESA9*, and *SsCESA12* were all suppressed in expression under cold stress, with their levels being the lowest at 6 hours (**Figure 7A**). When plants are subjected to heat stress, only *SsCESA10(t)*, *SsCESA3*, and *SsCESA12* are rapidly induced for expression at 6 hours. The expressions of the remaining genes are inhibited, reaching their lowest levels at 12 hours, except for *SsCESA4* (**Figure 7B**). Under salt stress, all *SsCESA* genes except *SsCESA2* were repressed (**Figure 7C**). These results indicate that the *SsCESA* gene family displays distinct expression patterns in response to cold, heat, and salt stresses.

### 2.7 Subcellular localization of SsCESA genes

**Figure 8:**
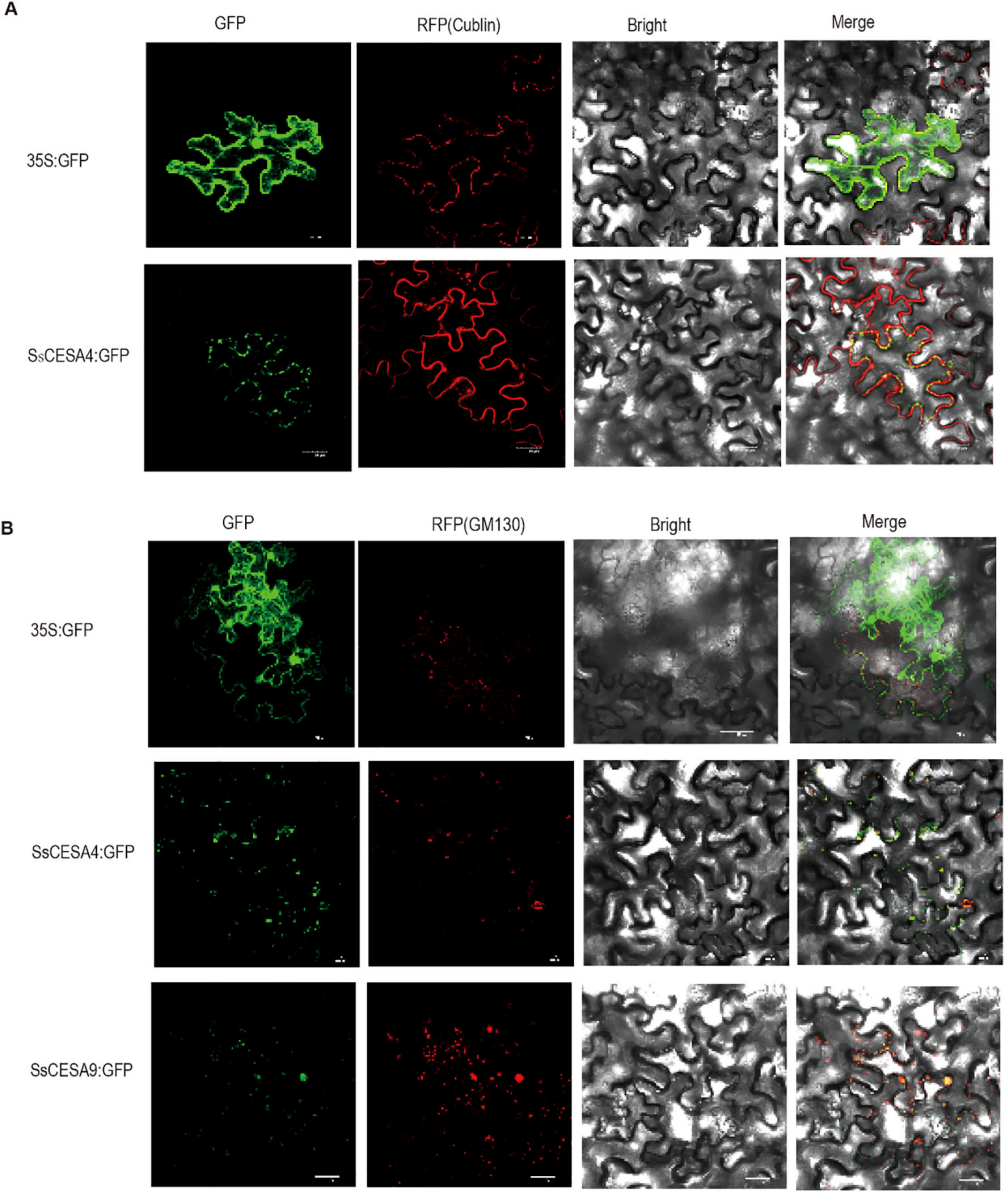
Subcellular localization of the SsCESA4 and SsCESA9 proteins in tobacco leaves. **(A)** 35s: GFP and SsCESA4: GFP co-localization with the cell membrane marker cubilin, respectively. **(B)** 35s: GFP, SsCESA4: GFP, and SsCESA9: GFP co-localization with golgi marker, respectively.

To further validate the subcellular localization of SsCESA proteins, we cloned *SsCESA4* and *SsCESA9* and performed transient transformation in tobacco leaves. We used the membrane protein marker “cubilin” to co-localize with SsCESA4-GFP. SsCESA4-GFP showed a punctate distribution, mainly on the cell membrane (**Figure 8A**). CESA proteins were reported to form complexes called CSCs within the endoplasmic reticulum or the Golgi apparatus (McFarlane et al., 2014). We conducted co-localization experiments of SsCESA4-GFP and SsCESA9-GFP with a Golgi marker, GM130, which showed that both proteins are located in the Golgi apparatus (**Figure 8B**). This supports the idea that cellulose synthase (CESA) complexes are likely to form in the endoplasmic reticulum (ER) and/or the Golgi apparatus. CESAs are also found in small post-Golgi structures (small CESA compartments, smaCCs) and are eventually transported to the plasma membrane, where cellulose is ultimately synthesized (Polko and Kieber, 2019; McFarlane et al., 2021).

### 2.8 SsNST1 binds to the SNBE sites in the promoters of *SCW*-*CESA* genes

**Figure 9:**
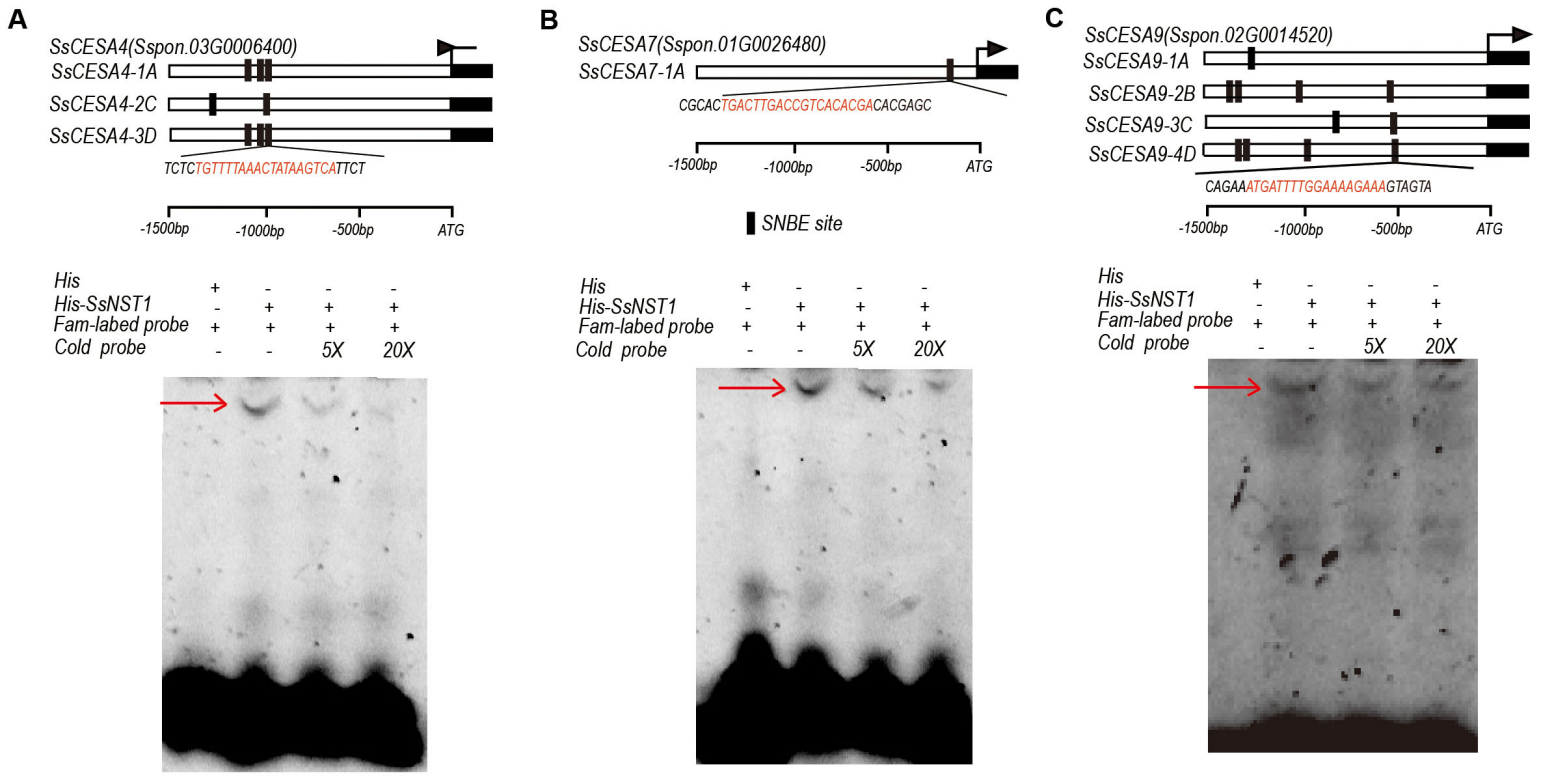
SsNST1 binds to the SNBE sites in the promoters of *SCW*-*CESA* genes **(A)** The promoters of *SsCESA4*. **(B)** The promoters of *SsCESA7*. **(C)** The promoters of *SsCESA9*. The dark rectangles indicate the locations and the sequences of the SNBE sites in the promoters of *SsCESA4*, *SsCESA7*, and *SsCESA9*, detected by PlantPAN 4.0. An EMSA assay to test the binding of SsNST1 to the target genes.

SNBE sites were identified within the promoter sequences of nearly all *SsCESA* members (**Figure 5D**). Seven, one, and eleven SNBE sites were identified in the promoters of *SsCESA4*, *SsCESA7*, and *SsCESA9*, respectively (**Figure 9**). The SNBE sites are binding sites associated with the secondary cell wall for NAC transcription factors. We selected one SNBE site from each of the three genes for validation. EMSA experiments demonstrated that NST1 can bind to the promoters of *SsCESA4*, *SsCESA7*, and *SsCESA9* via the SNBE sites (**Figure 9**).

## 3 Discussion

Sugarcane is a vital sugar and bioenergy crop, contributing to over 80% of global sugar production and serving as a key feedstock for renewable energy solutions such as ethanol and bioelectricity (Leal and Nogueira, 2014). Cellulose, as the primary component of the cell wall, assumes a vital role in plant growth and development. *CESA* genes are essential members of the *CESA/CSL* supergene family and are involved in cellulose biosynthesis (Li et al., 2014; Polko and Kieber, 2019). Among its progenitors, *S. spontaneum* contributes approximately 20% of the genetic background to commercial sugarcane (Glassop et al., 2021). The *CESA* genes have been predominantly investigated in model plants, while few in-depth studies have been conducted on other non-model plants (Pear et al., 1996; Richmond, 2000; Zhang et al., 2021a, 2021). *CESA* gene family members have been reported in economic crops and forest trees in recent years (Huang et al., 2022; Liu et al., 2022; Niu et al., 2023; Sod et al., 2023; Wang et al., 2023). However, despite the release of genomic information for *S. spontaneum* and for other sugarcane types, knowledge about the *SsCESA* genes remains limited.

We identified 30 members of the *SsCESA* gene family in the sugarcane (AP85-441) genome through HMM searching (**Table 1**). Given the tetraploid genome of AP85-441, 15 genes were discovered. Meanwhile, *SsCESA12*(*Sspon.08G0005550*) is unique to *S. spontaneum* and does not cluster with any members of rice or *Arabidopsis*. Since AP85-441 (*S. spontaneum*) is a tetraploid, there are usually one to four corresponding alleles for each of the typical *CESA* genes, such as *SsCESA4* (*Sspon. 03G0006400*), *SsCESA7*(*Sspon.01G0024680*), *SsCESA9* (*Sspon.02G0014520*), and *SsCESA12*(*Sspon.08G0005550*). This indicates that the major *CESA* genes are conserved in *S. spontaneum*, although the number of alleles does not match the genome ploidy. Through intraspecific synteny analysis, it was found that there are no tandem duplication events among the *SsCESA* gene family and 13 pairs of segmental duplication events among *SsCESA1, SsCESA3*, and *SsCESA8*. This indicates that the genes associated with the primary cell wall in *S. spontaneum* have undergone expansion through segmental duplication. The members of the *SsCESA* gene family exhibit the highest number of collinearity events with those of maize. This is associated with the fact that both *S. spontaneum* and maize are classified within the *Panicoideae* subfamily and the *Andropogoneae* tribe, and are polyploids (Grass Phylogeny Working, 2012).

Analysis of *cis-*acting elements in the promoter region reveals that *SsCESA* may be regulated by hormones and abiotic stresses. We identified many MYB transcription factor (TF) binding sites in the *SsCESA* promoter, potentially linked to the NAC-MYB-secondary cell wall regulatory network. It has been reported that the AtMYB46 protein directly binds to the promoters of the three *CESA* genes (*CESA4*, *CESA7*, and *CESA8*) both *in vitro* and *in vivo* (Kim et al., 2013). *PagMYB216* and *OsMYB61*, homologous genes of *AtMYB61*, stimulate the synthesis of three SCW *CESA* genes through their interaction with *GAMYB* motifs (Woodger et al., 2003; Huang et al., 2015; Wei et al., 2019). GhMYB7 and GhMYB46_D13 directly bind to three different cis-elements in the promoters of *GhCESA4*, *GhCESA7*, and *GhCESA8*, thereby regulating the synthesis of secondary cell wall cellulose in cotton fibers (Huang et al., 2021). Additionally, seven homologous pairs within the GhFSN (fiber secondary cell wall-related NAC1) subclade, including GhFSN1A, GhFSN2D, GhFSN3A, GhFSN4A, GhFSN5D, GhFSN6A, GhVND4D, and GhVNI1A, can form pairwise heterodimers that work together to activate *GhCESA* genes and promote cellulose biosynthesis (Chen et al., 2025). Moreover, we detected SNBE sites and a W-box in the *SsCESA* promoter, suggesting that NAC and WRKY TFs may directly regulate *SsCESA*. It has been demonstrated that AtNST2 can bind to the promoter of *AtCESA4* (Taylor et al., 2003; Ohashi-Ito et al., 2010). EMSA experiments demonstrated that SsNST1 can directly bind to the promoters of *SsCESA4*, *SsCESA7*, and *SsCESA9* (**Figure 9**). The expression levels of *AtCESA7 (IRX3)* and *AtCESA4 (IRX5)* are downregulated in the *atnst1*, *atnst3* double mutant (Mitsuda et al., 2007). MANAC1, which is the homolog of SND2, NST1 and SND1, can directly bind to and activate the *MACESA6B* and *MaCESA7* (Yin et al., 2024). All these results indicate that NAC transcription factors can activate the expression of downstream *CESA* genes. Therefore, we speculate that SsNST1 activates the expression of *SsCESA4*, *SsCESA7* and *SsCESA9*. However, to verify our speculation, a large number of *in vivo* and *in vitro* experiments as well as the construction of SsNST1 genetic materials are still needed. Meanwhile, more experimental evidence is required to confirm that WRKY TFs can directly regulate the *SsCESA* gene.

Co-expression patterns can reveal networks of functionally related genes, providing a deeper understanding of the processes involved in the production of multiple gene products (Wang et al., 2019). The *SsCESA* genes showed higher expression in mature stems than in leaves or leaf roll tissues (**Figure 6C**), suggesting a potential role in regulating biological processes related to stem development. This result aligns with the finding that *OsCESA* exhibits a relatively high expression level in rice stems, and the discovery that the *SbCESA* genes related to the primary and secondary cell walls reach their peak in sorghum stems at 75 days (Shakoor et al., 2014; Wang et al., 2016). Thus, the *CESA* genes in plants are essential for cellulose synthesis, which helps maintain the mechanical strength of the stem. Notably, through homology alignment with rice and *Arabidopsis thaliana*, as well as transcriptomic co-expression analysis, we indicate that *SsCESA4*, *SsCESA7*, and *SsCESA9* are associated with the secondary cell wall, whereas *SsCESA1*, *SsCESA3*, and *SsCESA8* may be related to the primary cell wall. Since primary and secondary growth are vital stages in plant development, secondary growth involves lignin synthesis and the activation of secondary metabolic pathways. Understanding the roles of various CESA members in these processes is essential for the next step in understanding biomass and yield formation in sugarcane. This is especially important for future research, as deleting or mutating primary cell wall genes may lead to events such as plant death. Therefore, future studies can use secondary cell wall-related genes as markers (such as *SsCESA4*, *SsCESA7*, and *SsCESA9* in this study) to examine how cell wall biosynthesis is regulated in sugarcane.

Abiotic stress, such as salinity, drought, and extremes of hot and cold temperatures, is believed to contribute to as much as 60% of yield reductions across multiple crop species (Ranjan et al., 2025). Cellulose is the primary structural component of plant cell walls and is the most prevalent biopolymer on the planet. Under cold stress, *SsCESA1* - *SsCESA4* could be activated (**Figure 7A**). *MaCESA7* and *MaCESA6B* were activated, resulting in an enhanced cellulose content during low-temperature stress (Yin et al., 2024). As an essential transcription factor in the cold regulatory pathway, the relationship between CBF (C-repeat Binding Factor) and *CESA* gene expression has not yet been reported. More experiments are needed for future verification. Most SsCESAs were downregulated under abiotic stress. Both rice and *Miscanthus* exhibited higher cellulose content in frost-sensitive genotypes compared to frost-tolerant ones (Domon et al., 2013; Xu et al., 2020). Meanwhile, mutations or RNA interference in the cellulose synthase gene can reduce cellulose levels and increase salt stress resistance (Li et al., 2017; Ye et al., 2021). However, ZmRR1 positively regulates the expression of *ZmDREB1* and *CESA* genes, thereby enhancing chilling tolerance (Zeng et al., 2021). Under heavy metal stress, *CESA* can enhance tolerance by increasing cellulose synthesis via hormone signaling (Kesten et al., 2017). The roles of cell wall-related genes in stress and hormone responses are a valuable area of study, from CSLD4’s salt tolerance to brittle stem genes, reported in model plants *Arabidopsis* and Rice (Ma et al., 2021; Zhao et al., 2022; Xu et al., 2023). When stressed, the variation in cellulose content makes the cell wall more plastic, allowing it to resist external changes. The findings on *SsCESA* genes and stress responses in sugarcane are worth exploring further.

## 4 Conclusions

This study for the first time deciphered the phylogeny, gene structure, *cis*-regulatory elements, and expression patterns of the *SsCESA* gene family in sugarcane (*S. spontaneum*). A total of 30 *SsCESA* genes were identified and distributed across 18 chromosomes. *SsCESA* genes exhibited extremely high expression levels in the stems and transitional parts of leaves. Moreover, *SsCESA4*, *SsCESA7*, and *SsCESA9* are mainly expressed at higher levels in mature stems, whereas *SsCESA1, SsCESA3*, and *SsCESA8* are expressed at higher levels in tender tissues (the basal parts of the leaves and the leaves of seedlings), indicating their roles in PCW and SCW, respectively. qRT-PCR experiments show that almost all *SsCESA* genes respond rapidly to cold, salt, and heat stress, suggesting they play a key role under stress. In addition, the SCW CESA proteins were further investigated and found to be localized in the Golgi apparatus. Transcription factor SsNST1 can bind to the SNBE site in the promoters of all three SCW *CESA* genes (S*sCESA4*, *SsCESA7*, and *SsCESA9*). Our results offer insights into the functional analysis of *SsCESA* genes and their regulation. *SsCESA4/7/9* are closely associated with secondary cell wall and cellulose biosynthesis. In sugarcane energy, upregulating these genes boosts cellulose, lowering ethanol costs. Higher cellulose also strengthens stems, improving lodging resistance.

## 5 Materials and methods

### 5.1 Plant materials

Stem and leaf tissues were collected from 35-day-old sugarcane seedlings of *S. spontaneum* (SES-208, 2n = 8x = 64). Samples including leaf roll, leaf, top internode (internode 3), maturing internode (internode 6), and mature internode (internode 9) were harvested from both 9-month-old and 12-month-old mature *S. spontaneum* plants, following the previously established methodology (Zhang et al., 2016; Chen et al., 2017; Li et al., 2020).

The sampling material for the transcriptome of the leaf development gradient was taken from the second leaves of 11-day-old *S. spontaneum* seedlings and cut into 15 segments, each5 cm long, following the method below (Li et al., 2010, 2020).

The material for the circadian rhythm transcriptome data was collected from the middle 4 cm of the first leaf of *S. spontaneum* in the experimental field of Fujian Agriculture and Forestry University. Sampling was conducted every 2 hours (6:00, 8:00, 10:00, 12:00, 14:00, 16:00, 18:00, 20:00, 22:00, 24:00, 2:00, 4:00) from March 2nd to 3rd, 2017, during the sugar accumulation stage (Ming et al., 2015; Li et al., 2020).

For the eight organizations conducting RT-qPCR experiments, the young and mature leaves and maturing stems were all from the germplasm resource pool at Guangxi University. The young leaves were taken from the leaves that had not fully unfolded from the apical meristem, and the mature leaves were the first leaves. The young stems and mature stems were taken from the first internode and second internode of *S. spontaneum* (counting from the top of the biological end downwards), respectively. The young leaves were placed in the callus induction medium (MS + 3 mg/L 2,4-D + 6 g/L agar) and kept in the dark for 1 month. Samples were taken when small, granular protrusions appeared on the leaf surface; these were the callus samples. The stem segments with buds were cut into uniform small pieces and then hydroponically cultured in the dark. Samples of the buds and newly grown young roots were taken respectively after 2 days and 7 days. The seedlings were the plants that had been hydroponically cultivated for two weeks using the stems of *S. spontaneous*.

*S. spontaneous* (AP85-441) seedlings were used for cold, heat, and salt stress experiments. We used stems of sugarcane *S. spontaneum* that were collected from the field. The buds were preserved, and the stems were evenly cut into segments of equal length. After two weeks of hydroponic cultivation, seedlings with similar growth status and size were selected for stress treatment. For heat treatment, the seedlings were placed in an incubator maintained at 45 °C. During the cold treatment, the seedlings were placed in an incubator set at 6 °C for treatment. For the salt stress treatment, the seedlings were immersed in a 150 mmol/L sodium chloride solution. All samples were collected at 0-, 6-, 12-, and 24 hours post-treatment, respectively. Five seedlings were combined into one pooled sample for RNA extraction.

### 5.2 Identification of *SsCESA* gene family in *S. spontaneum*

The Hidden Markov models (HMM) of the Glycos_transf_2 domain (PF00535) and the Cellulose synthase domain (PF03552) were downloaded from the Pfam database (http://pfam.xfam.org/) (Finn et al., 2016). The entire protein sequences of *S. spontaneum* were searched using HMMER software (v3.0) with an E-value set to 1×10^−5^, yielding preliminary candidate sequences. Subsequently, the candidate sequences were analyzed in the CDD (https://www.ncbi.nlm.nih.gov/cdd/) and SMART (http://smart.embl.de/) databases to confirm the conserved domains, retaining protein sequences that contained the Ring and Cellulose synthase domains (Letunic et al., 2020). Sequences with non-typical conserved domains or incomplete structures were removed, resulting in the identification of the final gene members.

### 5.3 Phylogenetic analyses of SsCESA proteins

The phylogenetic trees were constructed based on the candidate protein sequences of *S. spontaneum*, as well as the CESA proteins of rice and *A. thaliana*. The evolutionary tree of SsCESA protein sequences was constructed using the maximum likelihood (ML) method in MEGA7 (Kumar et al., 2016). The alignment method was Clustal W, the Bootstrap value was 1,000, and the model used was JTT+G. As both *S. spontaneum* and rice are monocotyledonous plants and share a closer phylogenetic relationship, the naming of *SsCESA* in *S. spontaneum* is based on that of rice.

### 5.4 Analysis of motifs, conserved domains, and gene structures in the *SsCESA* gene family

The protein sequences of the SsCESA were uploaded to the MEME website (http://meme-suite.org/tools/meme) for the prediction of conserved protein motifs (Bailey et al., 2009). The number of conserved motifs was set to 30, and the length of the conserved motifs was set to range from 5 to 200 amino acids. The gene structure was depicted using the gff3 file in TBtools. Protein domains were obtained from the SMART website (https://smart.embl.de) (Letunic et al., 2020). Subsequently, a combined graph encompassing the gene structure, conserved motifs, and domains was generated in TBtools (Chen et al., 2023).

### 5.5 Chromosomal localization and synteny analysis of SsCESA genes

Utilizing the genome annotation file of *S. spontaneum*, the chromosomal locations of the *SsCESA* gene family were visualized using Tbtools (Chen et al., 2023).

To deeper into the intraspecific synteny of the S*sCESA* genes, the syntenic gene pairs of S*sCESA* were identified via MCscanX analysis (Wang et al., 2012). Subsequently, the synteny circos plot was visualized using the “Advanced Circos” module within the TBtools software (Chen et al., 2023).

To further investigate the interspecific evolutionary relationships of *SsCESA*, five representative species were selected: *Oryza sativa* (rice), *Glycine max* (soybean), *Sorghum bicolor* (sorghum), *Triticum aestivum* (wheat), and *Zea mays* (maize). Collinearity analysis was performed with SsCESA from *Saccharum spontaneum*. The collinearity relationships among species were analyzed and visualized using TBtools software (Chen et al., 2023).

### 5.6 Predicting *cis*-acting regulatory elements in the promoter regions of the *SsCESA* gene family

The promoter sequences of *SsCESA* genes, extending 2000 bp upstream from the translational initiation codon (ATG), were retrieved from the *S. spontaneum* L. genome. Hormonal responsiveness, stress responsiveness, and growth and development-related *cis*-acting elements within these promoter regions were identified using the PlantCARE database (https://bioinformatics.psb.ugent.be/webtools/plantcare/html/Menu.html) (Lescot et al., 2002).The SNBE site([TA]NN[CT][TCG]TNNNNNNNA[AC]GN[ACT][AT]), the W-box (TTGAC[C/T]**),** and the MYB binding site([T/C]AAC[T/G] or CAAC) were searched in the PlantPAN 4.0 (https://plantpan.itps.ncku.edu.tw/plantpan4/index.html) (Chow et al., 2024). Ultimately, Tbtools was employed for image visualization.

### 5.7 Investigation of *SsCESA* gene expression patterns in *S. spontaneum* using public RNA sequencing

Since the nomenclature of *S. spontaneum* genes in the v20190103 genome differs from that in the Sugarcane Genome Database (SGD https://sugarcane.gxu.edu.cn/), we aligned the protein sequences of SsCESA from the v20190103 genome in the SGD to obtain the corresponding gene IDs (Zhang et al., 2018). We retrieved the gene expression levels of *SsCESA* from three publicly available transcriptome datasets on the SGD website, specifically those related to leaf development, circadian rhythm, and growth and development. The average value of the three replicates in the transcriptome data was calculated. Then, the data were subjected to logarithmic transformation and normalization procedures. Finally, we utilized the heatmap function in TBtools for visualization (Chen et al., 2023).

### 5.8 RNA extraction and RT-qPCR

Total RNA of seedlings and tissues was extracted using Trizol (R401, Vazyme, China). First-strand cDNA was generated using the TransScript All-in-One First-Strand cDNA Synthesis SuperMix for qPCR, which includes a one-step gDNA removal process (R323, Vazyme, China). Real-time qPCR was conducted using SYBR Green (Q713, Vazyme, China) on a Multicolor Real-Time PCR Detection System (Roche 480). The *GADPH* gene was chosen as an internal reference gene for normalization, and three biological replicates were employed (Ling et al., 2014). Relative gene expression levels were determined using the 2^-ΔΔCt method. A list of primers used in the RT-qPCR is provided in **Supplementary Table 11**.

### 5.9 Subcellular localization

Transient expression assays in *Nicotiana benthamiana* leaves were conducted using the agroinfiltration method, as outlined in Sparkes et al. To confirm subcellular localization, co-localization experiments were performed using specific organelle markers, including mCherry-Cubilin (a membrane marker). Fluorescence signals from GFP and mCherry were detected and recorded using a confocal laser scanning microscope (GFP: excitation at 488 nm, emission detected between 493–598 nm; mCherry: excitation at 587 nm, emission detected between 600–650 nm). A list of primers used in the subcellular localization is provided in **Supplementary Table 11**.

### 5.10 Electrophoretic Mobility Shift Assay

The entire coding sequence of SsNST1, tagged with a His tag, was cloned into the Escherichia coli strain BL21 to generate the His-SsNST1 recombinant protein. General Biol Company (Anhui, China) synthesized the 5′ 6-FAM-labeled probes. EMSA was performed as previously described (Gao et al., 2019). The DNA signals were detected using a fluorescence imaging system (Bio-Rad, California, USA). The probe DNA sequences are provided in **Supplementary Table 11**.

## Data Availability

The original contributions presented in the study are included in the article/**Supplementary Material**. Further inquiries can be directed to the corresponding author.
